# Ovarian remnant syndrome with paraintestinal ovarian serous cystadenofibroma arose 30 years after bilateral salpingo-oophorectomy: A case report

**DOI:** 10.1097/MD.0000000000031205

**Published:** 2022-11-04

**Authors:** Chin-Tzu Tien, Chiu-Hsuan Cheng, Dah-Ching Ding

**Affiliations:** a Department of Obstetrics and Gynecology, Hualien Tzu Chi Hospital, Buddhist Tzu Chi Medical Foundation, and Tzu Chi University, Hualien, Taiwan; b Department of Pathology, Hualien Tzu Chi Hospital, Buddhist Tzu Chi Medical Foundation, and Tzu Chi University, Hualien, Taiwan; c Institute of Medical Sciences, Tzu Chi University, Hualien, Taiwan.

**Keywords:** cystadenofibroma, laparo-endoscopic single-site surgery, laparoscopy, ovarian remnant syndrome, ovary

## Abstract

Ovarian cystadenofibroma is a relatively rare benign ovarian tumor. Ovarian remnant syndrome (ORS) is a rare complication of bilateral salpingo-oophorectomy (BSO). We report a rare case of ORS with paraintestinal ovarian serous cystadenofibroma that developed 30 years after total abdominal hysterectomy and BSO in a 73-year-old woman.

**Patient concerns:**

A 73-year-old woman complained of long-term lower abdominal discomfort.

**Diagnosis:**

She was diagnosed with a cystic lesion in the lower abdomen on transabdominal ultrasonography. Further diagnostic imaging and laboratory tests could not exclude a diagnosis of malignancy.

**Interventions:**

The patient underwent laparoendoscopic single-site surgery. We found one cystic lesion 5 cm in size with multiple septa that was adhered to the small bowel. We consulted a general surgeon for tumor resection. Dissection was performed and the specimen was then removed from the umbilical wound.

**Outcomes:**

Histopathological examination revealed an ovarian serous cystadenofibroma. The postoperative recovery was uneventful.

**Lessons:**

Patients with lower abdominal pain after a previous hysterectomy and BSO should be examined with transabdominal sonography for ORS.

## 1. Introduction

Ovarian cystadenofibroma is a relatively rare benign ovarian tumor. It may masquerade as a malignant ovarian tumor owing to the similarity of its features and findings on computed tomography (CT) or transvaginal/transabdominal ultrasonography.^[1]^

Ovarian remnant syndrome (ORS) was first reported in 1970^[2]^ and is a known complication of salpingo-oophorectomy. The authors reported that a functional ovarian remnant was found after an oophorectomy in felines. Neoplasia is a rare finding in ORS.^[3]^ The most common symptom of ORS is abdominal pain or an abdominal mass. The diagnosis and treatment of ORS is challenging for clinicians due to the nonspecific nature of the presenting symptoms, which may result in a missed or delayed diagnosis.

There are no reports of an ovarian cystadenofibroma following bilateral oophorectomy. We report a rare case of a 73-year-old woman who had ORS after bilateral salpingo-oophorectomy 30 years prior and was finally diagnosed with ORS with ovarian serous cystadenofibroma. The patient provided informed consent for her details to be used in this case report.

## 2. Case presentation

**Figure 1.**
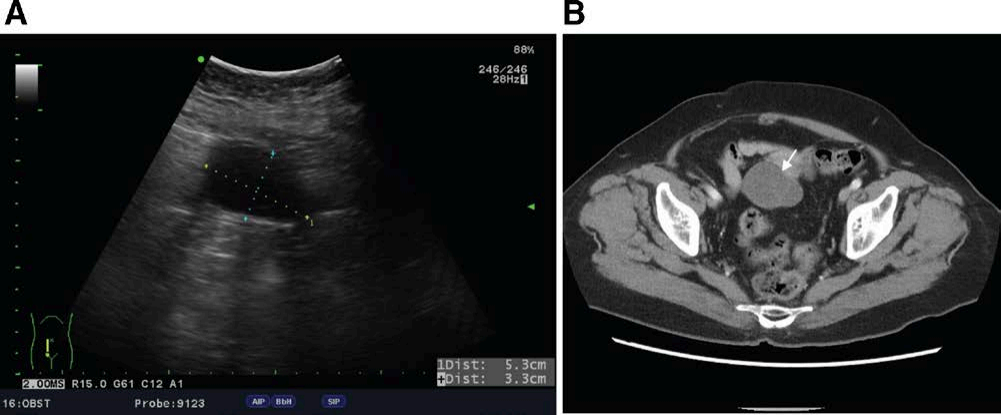
Tumor imaging. (A) Pelvic ultrasound showed a hypoechoic tumor 5.3 × 3.3 cm in size. (B) Computed tomography showed a tumor located in the intestinal region (arrow).

A 73-year-old woman, gravida 7, para 5 (all spontaneous vaginal deliveries), and 2 abortions experienced menopause 30 years ago after total abdominal hysterectomy (TAH) and bilateral salpingo-oophorectomy (BSO) due to leiomyoma. She had a medical history of hypertension for which she was taking a calcium channel blocker and type 2 diabetes mellitus which was under oral hypoglycemic control. In 2019 she had a lumboperitoneal shunt placed for hydrocephalus. She complained of dull lower abdominal pain for three years since 2021. Physical examination revealed a soft abdomen with tenderness in the right lower quadrant and normoactive bowel sounds. No remarkable findings were discovered on transvaginal ultrasound. Transabdominal ultrasonography revealed a cystic lesion 5.3 × 3.3 cm in the lower abdominal region and there was no free fluid in the cul-de-sac (Fig. 1A). The ORS was then suspected due to she had received TAH + BSO. Image study of CT of the pelvis revealed a multilocular cystic mass in the pelvis and among the bowel loops (Fig. 1B). Due to ORS may own cancer risk, a tumor marker panel assay was done. Laboratory test results showed carbohydrate antigen 19 to 9 levels of <0.8 U/mL (range 0–35 U/mL), mildly elevated carcinoembryonic antigen (CEA) of 3.5 ng/mL (range 0–3 ng/mL), and cancer antigen 125 (CA-125) levels of 70.4 U/mL (range 0–35 U/mL). Ovarian cancer risk is low but still existed. Therefore, conservative treatment was not considered. After discussing the cancer risk, she decided to receive a diagnostic laparoscopy. If malignancy was suspected during surgery, a frozen section would be sent.

**Figure 2.**
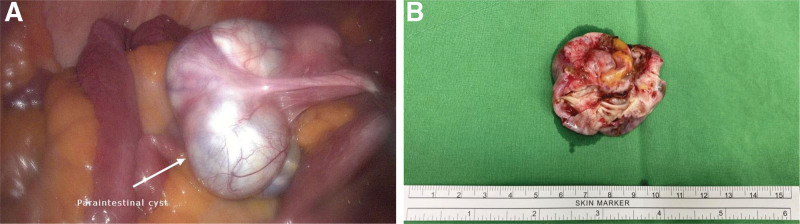
Laparoscopic image of the tumor. (A) The tumor adhered to the nearby intestinal surface. (B) Gross picture of the cystic tumor. A smooth cystic wall was noted.

**Figure 3.**
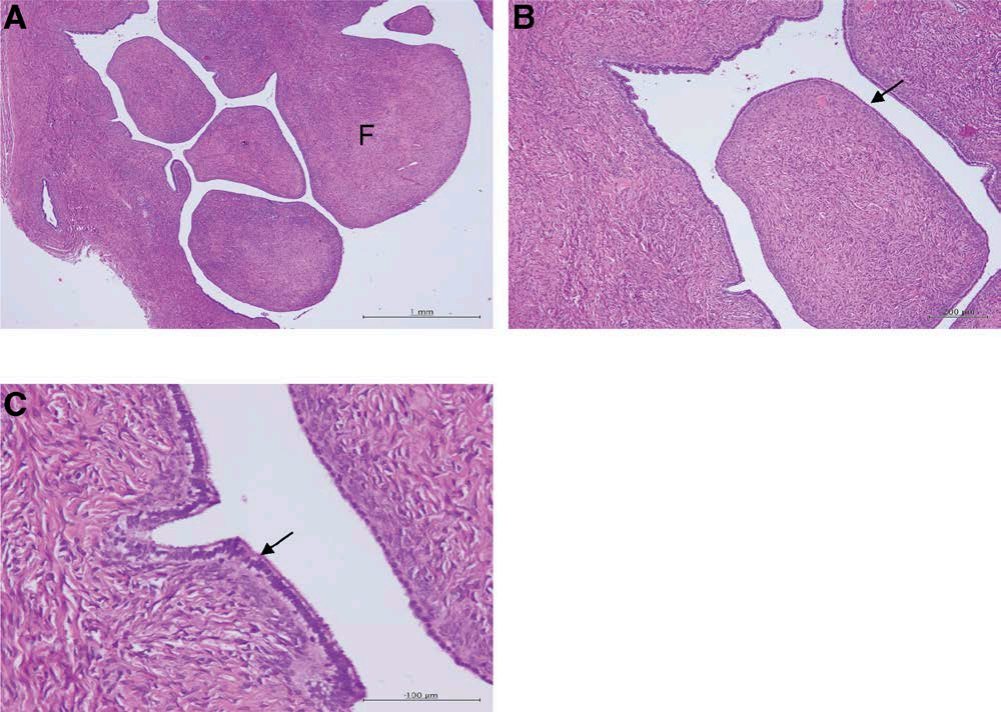
Histopathological picture of the tumor. (A) Papilla with fibrous stroma (F). Scale bar = 1 mm. (B) Papillae are covered by a single layer of tubal-type epithelium (arrow). Scale bar = 200 μm. (C) Bland tubal type epithelium with cilia (arrow). Scale bar = 100 μm.

The patient underwent diagnostic laparoendoscopic single-site surgery. A paraintestinal cyst with a smooth surface measuring 5 × 3 cm with omental adhesion to the anterior pelvic wall was found (Fig. 2A). We consulted a general surgeon for the enterolysis and tumor excision. After enterolysis, we removed the paraintestinal cyst through the umbilical port and dissected it from the intestine using monopolar coagulation. The small bowel serosa was torn after resecting the tumor and it was repaired using an interrupted suture with 3 to 0 silk. After dissecting the tumor, the specimen revealed that the ovarian cyst had a smooth inner surface (Fig. 2B). A benign tumor was considered then. Histopathology determined the diagnosis of ovarian serous cystadenofibroma (Fig. 3A–C). The postoperative course was uneventful, and the patient was discharged 2 days later.

## 3. Discussion

ORS usually results from unintended incomplete oophorectomy for any indication, with or without hysterectomy. The incidence of ORS is difficult to calculate because of its rarity. One cohort study showed that there was a 22% incidence of developing ORS in patients who received TAH and BSO during one to eight years of follow-up.^[4]^ It is believed to have a higher incidence in patients who had endometriosis, pelvic inflammatory diseases due to distorted anatomy, extensive adhesion, and increased vascularity, resulting in incomplete removal of the ovarian tissue.

Furthermore, patients with ORS may be at risk of ovarian cancer. Endometriosis is thought to be associated with the risk of ovarian cancer^[5,6]^ and ORS associated with cyclic pelvic pain is often suspected in endometriosis.^[7]^ Previous case reports showed that patients with ORS may have ovarian adenocarcinoma.^[8–10]^ Therefore, patients should be screened for ovarian cancer following oophorectomy based on risk factors, symptoms, and imaging studies.

Most patients with ORS have chronic pelvic pain or persistent cyclic pelvic pain, with or without findings of an abdominal mass. Even a small piece of ovarian tissue can respond to hormone stimulation with growth, cystic degeneration, or hemorrhage, leading to pain in premenopausal women. The diagnosis of ORS includes a history of oophorectomy with or without hysterectomy, pelvic examination, ultrasound, CT, or magnetic resonance imaging (MRI) studies. The diagnosis may be enhanced by measuring serum levels of follicle-stimulating hormone, luteinizing hormone, and estradiol.^[11]^ However, the definitive diagnosis is made by histologic examination of the tissue removed during surgery.

Ovarian serous cystadenofibroma is a rare benign ovarian tumor characterized by the presence of cystic structures lined by serous epithelial cells in a fibrotic stroma.^[12]^ It is hard to distinguish this type of benign ovarian tumor from malignancy due to the similarity of imaging features of the two conditions on ultrasonography, CT, or MRI. One study found that some ovarian serous cystadenofibromas had pure cystic lesions, while the rest had cystic components with thick or irregular septa. Solid components in the cystic tumors were correlated with fibrous stroma that occasionally produced a false-positive result for malignancy on imaging.^[1]^ Wasnik and Elsayes found the key MRI feature of ovarian cystadenofibroma to be the presence of low-signal intensity on T2-weighted images due to the fibrous tissue component and this may help in differentiating complex ovarian or adnexal lesions before surgery.^[13]^

Transvaginal ultrasonography is usually used during the routine follow-up of patients who have previously undergone TAH and BSO. However, in this case, transvaginal ultrasonography results were negative. Transabdominal sonography was performed and a cystic lesion was noted. Mild elevation of CEA and CA-125 levels were observed. Therefore, malignancy could not be ruled out. Surgery was scheduled and ovarian cystadenofibroma was diagnosed through histological examination of the resected specimen.

Conservative medical treatment or symptomatic temporary management of ORS have been suggested for patients without a histological diagnosis of ORS who are at high risk of surgical complications. Oral contraceptive pills, gonadotropin-releasing hormone analogs, and medroxyprogesterone may be used to suppress ORS. Pelvic radiation therapy for residual ovarian tissue is another effective treatment for some patients. However, treatment of a pelvic mass with medication or radiation therapy without a histological diagnosis of ORS may contravene medical practice as the possibility of malignancy has not been excluded from the differential diagnosis. Surgical excision by laparotomy or laparoscopy is the most widely accepted treatment, since complete excision of ovarian remnants is the best method to improve symptoms and avoid further corrective surgery. Furthermore, the pelvic mass can be diagnosed by histology directly without missing a potential malignancy. Laparoscopy seems to be as safe and effective as laparotomy for the removal of ovarian remnants, with better visualization, identification of anatomy, and less blood loss.^[4,14]^

In conclusion, a paraintestinal cyst of ovarian origin should be considered, even if the patient has a previous history of bilateral oophorectomy. Clinicians should be aware of this possible pathogenesis due to ORS and should use transabdominal or transvaginal sonography to examine patients who have lower abdominal pain after a previous hysterectomy and bilateral salpingo-oophorectomy.

## Author contributions

**Data curation:** Chin-Tzu Tien, Chiu-Hsuan Cheng.

**Investigation:** Chin-Tzu Tien.

**Methodology:** Chin-Tzu Tien, Chiu-Hsuan Cheng.

**Project administration:** Dah-Ching Ding.

**Supervision:** Dah-Ching Ding.

**Writing– Original draft:** Chin-Tzu Tien.

**Writing – Review & editing:** Dah-Ching Ding.
